# Na^+^/Ca^2 +^ Exchange and Pacemaker Activity of Interstitial Cells of Cajal

**DOI:** 10.3389/fphys.2020.00230

**Published:** 2020-03-18

**Authors:** Haifeng Zheng, Bernard T. Drumm, Mei Hong Zhu, Yeming Xie, Kate E. O’Driscoll, Salah A. Baker, Brian A. Perrino, Sang Don Koh, Kenton M. Sanders

**Affiliations:** Department of Physiology and Cell Biology, University of Nevada School of Medicine, Reno, NV, United States

**Keywords:** ANO1, Ca^2+^-activated Cl^–^ current, slow waves, smooth muscle, gastrointestinal motility

## Abstract

Interstitial cells of Cajal (ICC) are pacemaker cells that generate electrical slow waves in gastrointestinal (GI) smooth muscles. Slow waves organize basic motor patterns, such as peristalsis and segmentation in the GI tract. Slow waves depend upon activation of Ca^2+^-activated Cl^–^ channels (CaCC) encoded by *Ano1*. Slow waves consist of an upstroke depolarization and a sustained plateau potential that is the main factor leading to excitation-contraction coupling. The plateau phase can last for seconds in some regions of the GI tract. How elevated Ca^2+^ is maintained throughout the duration of slow waves, which is necessary for sustained activation of CaCC, is unknown. Modeling has suggested a role for Na^+^/Ca^2+^ exchanger (NCX) in regulating CaCC currents in ICC, so we tested this idea on murine intestinal ICC. ICC of small and large intestine express NCX isoforms. NCX3 is closely associated with ANO1 in ICC, as shown by immunoprecipitation and proximity ligation assays (PLA). KB-R7943, an inhibitor of NCX, increased CaCC current in ICC, suggesting that NCX, acting in Ca^2+^ exit mode, helps to regulate basal [Ca^2+^]_*i*_ in these cells. Shifting NCX into Ca^2+^ entry mode by replacing extracellular Na^+^ with Li^+^ increased spontaneous transient inward currents (STICs), due to activation of CaCC. Stepping ICC from −80 to −40 mV activated slow wave currents that were reduced in amplitude and duration by NCX inhibitors, KB-R7943 and SN-6, and enhanced by increasing the NCX driving force. SN-6 reduced the duration of clustered Ca^2+^ transients that underlie the activation of CaCC and the plateau phase of slow waves. Our results suggest that NCX participates in slow waves as modeling has predicted. Dynamic changes in membrane potential and ionic gradients during slow waves appear to flip the directionality of NCX, facilitating removal of Ca^2+^ during the inter-slow wave interval and providing Ca^2+^ for sustained activation of ANO1 during the slow wave plateau phase.

## Introduction

Interstitial cells of Cajal provide the pacemaker activity responsible for electrical slow waves in gastrointestinal (GI) muscles (Ward et al., 1994; Huizinga et al., 1995; Dickens et al., 1999; Zhu et al., 2009). Conduction of slow waves to SMCs causes depolarization (Cousins et al., 2003), activation of voltage-dependent Ca^2+^ channels (Mitra and Morad, 1985; Droogmans and Callewaert, 1986; Langton et al., 1989), Ca^2+^ entry, and initiation of contraction (Ozaki et al., 1991; Vogalis et al., 1991). The occurrence of slow waves results in phasic contractile patterns in most areas of the GI tract. A major conductance responsible for slow wave currents in ICC is due to Ca^2+^-activated Cl^–^ channels (CaCC) encoded by *Ano1* (Hirst et al., 2002; Hwang et al., 2009; Zhu et al., 2009). While it appears that Ca^2+^ entry and Ca^2+^ release from stores via IP_3_ and ryanodine receptors are important sources of Ca^2+^ for the activation of slow wave in ICC (Suzuki et al., 2000; Zheng et al., 2014; Zhu et al., 2015), the mechanisms required to maintain openings of CaCC for the relatively long durations of slow waves are not understood. For example, slow waves recorded by direct impalement of ICC in the small intestine of the mouse, depolarize to about −10 mV (estimated *E*_*Cl*_) during the upstroke phase, and depolarization is sustained near that potential for at least 1 s (plateau phase) or more in ICC from other regions (Dickens et al., 1999; Kito and Suzuki, 2003; Kito et al., 2005). The plateau phase of slow waves has been attributed to clusters of Ca^2+^ transients in ICC (Drumm et al., 2017) that cause sustained activation of CaCC (Hirst et al., 2002; Kito and Suzuki, 2003), but the mechanism(s) responsible for sustaining Ca^2+^ release events during the plateau phase are unknown.

Na^+^/Ca2^+^ exchanger is typically considered a Ca^2+^ extrusion transporter, but the exchanger is capable of bi-directional movement of ions depending upon the membrane potential and the transmembrane gradients for Ca^2+^ and Na^+^ (Blaustein and Lederer, 1999). When NCX favors Ca^2+^ entry into cells, rather than extrusion, this has been called the “Ca^2+^ entry mode.” Ca^2+^ entry via the Ca^2+^ entry mode of NCX has been reported in various cells (Leblanc and Hume, 1990; Luther et al., 1992), and pacemaker activity in urethra interstitial cells and ICC in the small intestine have been suggested to be linked to NCX operating in Ca^2+^ entry mode (Bradley et al., 2006; Lowie et al., 2011; Drumm et al., 2015). The function of NCX and the proximity between NCX proteins and ANO1 channels in microdomains within cells may be contributing factors in activation and deactivation of CaCC in ICC and in the sustained activation of CaCC during the slow wave plateau phase, as recent modeling of slow waves suggests (Youm et al., 2019).

Ultrastructural studies of ICC document regions of plasma membrane that are closely associated with ER in ICC, and it is possible that dynamic changes in ionic concentrations due to release of Ca^2+^ from ER and transmembrane fluxes of various ions into and out of the restricted volumes created by such structures [referred to as microdomains or “pacemaker units” (Sanders et al., 2006)] are fundamental to generation of pacemaker currents. The underlying pacemaker event in ICC is the stochastic generation of STICs, which lead to STDs of membrane potential (Edwards et al., 1999; van Helden et al., 2000; Zhu et al., 2009). As in other cells (Hogg et al., 1993; Bao et al., 2008), STICs in ICC are likely due to transient Ca^2+^ release events into the microdomains between the plasma membrane and ER. Openings of CaCC are synchronized by voltage-dependent Ca^2+^ conductances to create the large amplitude inward currents responsible for slow waves in ICC (Zhu et al., 2009).

In the present study we evaluated expression of *Slc8a1-3* (genes encoding NCX1-3) in ICC isolated from the murine colon and small intestine and purified by FACS. We compared expression of *Slc8a1-3* transcripts in ICC and in the mixed cell population obtained from enzymatic dispersion of muscles. We found that NCX3 is associated with ANO1 channels, and we evaluated the function of NCX in activating and sustaining activation of CaCC during slow wave currents using the patch clamp techniques on single ICC and imaging of Ca^2+^ transients in intact networks of pacemaker ICC.

## Materials and Methods

### Animals

C57BL/6 (Charles River Laboratories, Wilmington, MA, United States) and *Kit*^*copGFP/*+^ mice, described previously (Zhu et al., 2009), were used for most experiments. Colon SMCs, used for control studies of L-type Ca^2+^ channels, were obtained from C57BL/6 mice (2 and 3 months old; Jackson laboratory, Sacramento, CA, United States). For imaging experiments Ai95 (RCL-GCaMP6f)-D (GCaMP6f mice) and their wild-type siblings (C57BL/6) were purchased from the Jackson Laboratory (Bar Harbor, MN, United States). B6. c-Kit^+/Cre–ERT2^ (Kit-Cre mice) were gifted from Dr. Dieter Saur of the Technical University Munich, Germany. GCaMP6f mice were crossed with Kit-Cre mice, and the resulting offspring are referred to as Kit-Cre-GCaMP6f mice throughout the manuscript. These mice were injected with tamoxifen at 6–8 weeks of age to induce Cre Recombinase and activate expression of GCaMP6f. Tamoxifen (Sigma T5648; 80 mg) was dissolved in 800 μL of ethanol (Pharmco-Aaper 200 Proof – Absolute, Anhydrous) by vortexing for 20 min. Then 3.2 ml of Safflower (generic) was added to create solutions of 20 mg/ml, which were then sonicated for 30 min prior to injection. Mice were injected (Intraperitoneal injection; IP) with 0.1 ml of tamoxifen solution (2 mg tamoxifen) for three consecutive days. Mice were used for experiments 10 days after the first injection. Expression of GCaMP6f was confirmed by genotyping and imaging.

On the days of experiments animals were anesthetized with isoflurane (Aerrane; Baxter, Deerfield, IL, United States) prior to decapitation and then small intestines and colon were removed. All animals used and the protocols performed throughout this study were in accordance with the National Institutes of Health Guide for the Care and Use of Laboratory Animals. The institutional Animal Use and Care Committee at the University of Nevada approved all procedures used in the breeding and killing of animals.

### Isolation of Cells

Interstitial cells of Cajal were isolated from Kit^*copGFP/*+^ mice, as described previously (Zhu et al., 2009). Briefly, jejunal and colon muscles were cut into small strips (about 1 cm) and put in Ca^2+^-free Hanks’ solution for 20 min. The muscles were incubated in a Ca^2+^-free enzyme solution containing (per ml): collagenase (Worthington Type II, 1.3 mg), bovine serum albumin (Sigma, St Louis, MO, United States, 2 mg), trypsin inhibitor (Sigma, 2 mg) and ATP (0.27 mg). Cells were plated onto coverslips coated with murine collagen (2.5 mg ml^–1^, BD Falcon, Franklin Lakes, NJ, United States). The cells were allowed to stabilize for 2 to 6 h at 37°C in an incubator (atmosphere 95% O_2_–5% CO_2_) in culture media (Clonetics, San Diego, CA, United States) supplemented with 2% antibiotic–antimycotic (Gibco, Grand Island, NY, United States) and stem cell factor (5 ng ml^–1^, Sigma).

To isolate SMCs from proximal colon (Koh et al., 1997), the organs were opened, and mucosa and submucosa were removed. The remaining muscles were cut into small strips and incubated in a Ca^2+^-free enzyme solution containing (per ml): collagenase (Worthington Type II, 4 mg), bovine serum albumin (8 mg), trypsin inhibitor (8 mg), papain (1 mg), and L-dithiothreitol (Sigma-Aldrich, MO; 0.15 mg). After incubation in the enzyme solution for 30 min, SMCs liberated by trituration and stored in Ca^2+^-free Hanks’ solution at 4°C for no more than 6 hrs.

### Collection of Cells for Analysis of Gene Expression

Interstitial cells of Cajal from *Kit*^*copGFP/*+^ mice were purified by fluorescence-activated cell sorting (FACS; BectonDickinson FACSAria) using 488 nm excitation and a 530/30-nm bandpass filter for GFP, as previously described (Peri et al., 2013). Total RNA was isolated from ICC and the mixed cell population obtained after dispersion (pre-unsorted cells) using illustra RNAspin Mini RNA Isolation kit (GE Healthcare, Little Chalfont, United Kingdom), and first-strand cDNA was synthesized using SuperScript III (Invitrogen, Carlsbad, CA, United States), according to the manufacturer’s instructions. PCR was performed with specific primers using AmpliTaq Gold PCR Master Mix (Applied Biosystems, Foster City, CA, United States). Primer sequences are provided in Table 1. PCR products were analyzed on 2% agarose gels and visualized by ethidium bromide. Quantitative PCR (qPCR) was performed with the same primers used for PCR using Syber green chemistry on the 7300 Real Time PCR System (Applied Biosystems). Regression analysis of the mean values of eight multiplex qPCRs for the log10 diluted cDNA was used to generate standard curves. Unknown amounts of messenger RNA (mRNA) were plotted relative to the standard curve for each set of primers and graphically plotted using Microsoft Excel. This gave transcriptional quantification of each gene relative to the endogenous hypoxanthine guanine phosphoribosyltransferase (*Hprt*) standard after log transformation of the corresponding raw data.

**TABLE 1:** List of primers used for PCR and qPCR.

Gene name	Primer sequence	Product length (bp)	Accession number
*Hprt*	F – GACTTGCTCGAGATGTCATGAAGGAGAT	198	NM_013556
	R – TGTCCCCCGTTGACTGATCATTACAGTA	(Exons 3–4)	
*Slc8a1* (NCX1)	F – TGC AGA CCG GTT TAT GTC CT	123	NM_011406
	R – TTC GAC ACA GTC TCG TTC CA	(Exons 13–14)	
*Slc8a2* (NCX2)	F – CTG CGT TCC ACC CAC GGA GT	190	NM_148946
	R – GCT GGC GAA CGT GTC AGG GA	(Exons 40–41)	
*Slc8a3* (NCX3)	F – AGT GCA GGA GGG GAT GAG GAT G	159	NM_080440
	R – GGA GAC CAC GAA GCA GGC CC	(Exons 3–4)	

### Electrophysiological Recording

Interstitial cells of Cajal were identified as cells with green fluorescent protein using an inverted fluorescence microscope. The standard whole-cell patch clamp configuration was employed to record membrane currents (voltage clamp mode). Currents were amplified with an Axopatch 200B patch-clamp amplifier (Axon Instruments, Union City, CA, United States) and digitized with a 16-bit analog to digital converter (Digidata 1440A, Axon Instruments) and stored using pCLAMP software (version 10.2, Axon Instruments). Data were sampled at 4 kHz and filtered at 2 kHz using an eight-pole Bessel filter for whole-cell experiments. All data were analyzed using Clampfit (pCLAMP version, 10.2, Axon Instruments, United States) and Graphpad Prism (version 3.0, Graphpad Software Inc., San Diego, CA, United States) software.

L-type Ca^2+^ currents were recorded from SMCs using amphotericin perforated whole cell patch clamp configuration. The stock solution of 90 mg/ml Amphotericin B (Sigma) was dissolved with dimethyl sulfoxide (DMSO) using sonication and diluted to a final concentration of 250 μg/ml in the pipette solution. STIC amplitude was measured using threshold events detection analysis in Clampfit. Ten seconds of each recording were analyzed. The minimum threshold amplitude of STICs was set to 6 pA and the averaged amplitude of all events from one recording was displayed as *n* = 1. The number of events in 10 s was used to determine the frequency of STICs (counts per minute, cpm). The amplitudes of currents in each cell activated by KB-R7943 or SN-6 at 0 mV were normalized to its cell capacitance (current density, pA/pF). The tail currents of slow wave currents were obtained by repolarization from −40 to −80 mV. The durations of tail currents were measured from the peak amplitude of inward tail currents to the end of tail currents (time to return to initial holding current).

The external solution for whole-cell recordings was a Ca^2+^-containing physiological salt solution (CaPSS; see Solution I in Table 2). Two different internal solutions were used for present study: (1) *E*_*Cl*_ = 0 mV solution (Solution V) and (2) *E*_*C*__*l*_ = −40 mV solution (Solution VI). In some experiments, NaCl (0 and 20 mM) of external solution were replaced with equimolar LiCl (Solution II and III). For measurement of ANO1 currents expressed in HEK 293 cell, an external solution was Solution IV and internal solution was Solution VII. For Cav3.2 and L-type Ca^2+^ currents, external and internal solutions were Solution I and Solution V, respectively.

**TABLE 2:** The composition of pipette solutions and bath solution.

Solutions (mM)	I	II	III	IV	V	VI	VII
NaCl	140		20				
LiCl		140	120				
NMDGCl				150			147.4
KCl	5	5	5				
CaCl_2_	2	2	2	2			4.14
MgCl_2_	1.2	1.2	1.2	1			
Glucose	10	10	10				
CsCl					140	30	
Cesium aspartate						110	
MgATP					3	3	
NaGTP					0.1	0.1	
Creatine phosphate disodium					2.5	2.5	
EGTA					0.1	0.1	10
HEPES	10	10	10	10	10	10	10
Free Ca^2+^					<1 nM	<1 nM	100 nM

*Solutions I, II, III, and IV were adjusted to pH 7.4 with Tris and solutions V, VI, and VII were adjusted to pH 7.2 with Tris.*

### Imaging and Analysis of Ca^2+^ Transients

Flat sheets of jejunum muscles were pinned to the bottom of a 60 mm dish coated with Sylgard elastomer (Dow Corning, Midland, MI, United States), with the longitudinal muscle side facing upward. The dish was continuously perfused with warmed KRB solution (37°C) for 1 h before experimentation. Following this equilibration period, Ca^2+^ imaging was performed *in situ* with an Eclipse E600FN microscope (Nikon Inc., Melville, NY, United States) equipped with a 60 × 1.0 CFI Fluor lens (Nikon instruments Inc., NY, United States). GCaMP6f was excited at 488 nm (T.I.L.L. Polychrome IV, Grafelfing, Germany). The pixel size using this acquisition configuration was 0.225 μm. Image sequences were collected at 33 fps with TILLvisION software (T.I.L.L. Photonics GmbH, Grafelfing, Germany). Imaging was performed in the presence of nicardipine (100 nM) to reduce contractile movements and any residual movements were stabilized digitally with Volumetry software prior to analysis of Ca^2+^ transients. For experiments involving pharmacological treatments, control video sequences were collected for 20–30 s, and then KRB solution containing the drug concentration to be tested was perfused into the bath for 12–15 min before another 20–30 s period of imaging was performed.

Ca^2+^ transients in myenteric ICC (ICC-MY) were quantified using particle (PTCL) analysis, as described previously (Drumm et al., 2017, 2018, 2019; Zheng et al., 2018). Briefly, movies were imported into custom software (Volumetry G8d) and motion stabilized to minimize residual motion artifacts. A differential (Δ*t* = ± 66–70 ms) and Gaussian filter (1.5 × 1.5 μm, StdDev 1.0) was applied to distinguish Ca^2+^ transients from the background. A particle analysis routine was applied by using of a flood-fill algorithm that marked the structure of all adjoining pixels that had intensities above the threshold. Ca^2+^ transient PTCLs were brighter and larger than noise particles. The threshold at which noise particles emerged and reduced the average particles size was thresholded and then valid Ca^2+^ PTCLs, above this threshold, were saved as a coordinate based PTCL movie. Any remaining noise in the PTCL file was removed by only including PTCLs > 6 μm^2^ (diameter ∼2 μm or smaller) in analysis. Ca^2+^ transients in ICC-MY occurred in temporal clusters (Ca^2+^ transient clusters, CTCs) of multiple firing sites in the ICC-MY network firing asynchronously within a ∼1 s window as previously described (Drumm et al., 2017, 2018, 2019). The frequency of CTCs was quantified as the number of CTCs per minute and the duration of CTCs was measured as 90% duration.

### Co-immunoprecipitation Assay

Co-immunoprecipitation (Co-IP) studies were performed to investigate possible protein-protein interactions between NCX3 and ANO1. Small intestine smooth muscle tissues were snap-frozen in liquid nitrogen and stored at −80°C after removal of the mucosa and sub-mucosa. Muscles were homogenized in lysis buffer (mM: 50 Tris–HCl pH 8, 60 β-glycerophosphate, 25 Na-pyrophosphate, 100 NaF, 5 EGTA, 1 MgCl_2_, 1 dithiothreitol, and protease inhibitor tablet; Roche, Indianapolis, IA, United States), centrifuged at 16,000 × *g* at 4°C for 10 min. The 16,000 × *g* supernatant was centrifuged at 100,000 × *g* for 1 h to obtain the membrane fraction (100,000 × *g* pellet). The pellet was resuspended into IP/lysis buffer (mM: 50 Tris–HCl pH 8, 60 β-glycerophosphate, 25 Na-pyrophosphate, 100 NaF, 5 EGTA, 1 MgCl_2_, 1 dithiothreitol, 0.5% NP40, and protease inhibitor tablet), and protein concentrations were determined by a Bradford assay using bovine g-globulin as the standard. ANO1, NCX3, and rabbit IgG antibodies (AB-105C) (R&D Systems, Minneapolis, MN) (5 μg) were crosslinked to Protein-A/G-magnetic beads using the Pierce Crosslink Magnetic IP/Co-IP kit according to the manufacturer’s instructions (ThermoFisher Scientific, Waltham, MA, United States). Membrane fractions (500 μg) were incubated with either rabbit anti-NCX3 specific antibody (AP12808b) (Abcepta, San Diego, CA, United States), or rabbit anti-ANO1 antibody (ab64085) (Abcam, Cambridge, MA, United States) overnight with rotation at 4°C. Captured proteins were eluted with low pH buffer. Eluted proteins were analyzed using the anti-NCX3 or the anti-ANO1 antibodies with ProteinSimple Wes (Li et al., 2018).

### Proximity Ligation Assay (PLA)

Isolated ICC were incubated on slides coated with murine collagen (2.5 mg ml^–1^, BD Falcon, Franklin Lakes, NJ, United States) for 4 h, fixed in 4% paraformaldehyde for 4 min, and then permeabilized and blocked with PBS containing 0.2% tween-20 and 1% bovine serum albumin for 10 min at room temperature. PLA was performed following the manufacturer’s instructions (Duolink Detect, Olink Bioscience, Sweden). The fixed and permeabilized cells were incubated with goat anti-NCX3 antibody (1:200 dilution) (sc-48896; Santa Cruz Biotechnology, Santa Cruz, CA, United States) for 1 h, at room temperature, washed three times with PBS and then incubated with the rabbit anti-ANO1 antibody (1:200 dilution) for 1 h at room temperature. The slides were washed three times with PBS and then incubated with the Duolink minus-anti rabbit IgG and plus-anti goat IgG secondary antibodies (1:5 dilution) at 37°C for 1 h, followed by the ligation and amplification reactions (Duolink detection kit red, ex.598/em.634). Finally, mounting medium with DAPI was used. The slides were examined using a LSM510 Meta (Zeiss, Jena, Germany) or Fluoview FV1000 confocal microscope (Olympus, Center Valley, PA, United States). Confocal micrographs are digital composites of the Z-series of scans (0.5 μm optical sections of 7 μm thick sections). Settings were fixed at the beginning of both acquisition and analysis steps and were unchanged. Final images were constructed using FV10-ASW 2.1 software (Olympus). As a negative control (two proteins not in close proximity), primary rabbit anti-ANO1 and mouse anti-myosin light chain (sc-48414) (Santa Cruz Biotechnology, Santa Cruz, CA, United States) antibodies were used, followed the by plus-anti rabbit IgG and minus-anti mouse IgG secondary antibodies, and the ligation and amplification reactions. The number of PLA spots per cell was divided by cell area, and these values were normalized to obtain PLA density values. Cell area was calculated with Image J software (NIH).

### Expression of ANO1 and CACNA1H (Ca_*v*_3.2) in HEK 293 Cells

An expressed sequence tag (IMAGE Consortium cDNA clone number 30547439) homologous to murine *Ano1* (A variant) was subcloned into pcDNA3.1 (Invitrogen). *Ano1* was ligated into the pmKate2-N vector (Evrogen, Moscow, Russia), containing the red fluorescent protein mKate2. The *Ano1* stop codon was removed and *Eco*RI and *Apa1* restriction sites were placed at the termini by PCR, so the *Ano1* transcript was inserted in frame and resulted in a plasmid encoding a C-terminal mKate2 tagged ANO1 fusion protein. An *Ano1* splice variant, AC, that is expressed in gastrointestinal muscles (Hwang et al., 2009), was generated by inserting the 12-nucleotide ‘C’ segment into the *Ano1* coding region using a QuickChange XL site-directed mutagenesis kit (Agilent Technologies). The plasmids were sequenced by the Nevada Genomics Centre to confirm insertion of the alternative exon. *Ano1* (AC splice variant) was expressed in human embryonic kidney (HEK) 293 cells (American Type Culture Collection, Manassas, VA). HEK 293 cells were seeded in 12-well plates and maintained in DMEM (Gibco) medium with FBS (10%, v/v, Gibco), penicillin–streptomycin (1%, v/v, Gibco) and glutamax (1%, v/v; Gibco). The plasmid containing *Ano1* tagged with mKate2 (0.5 μg/well) was transfected into cells using 1.5 μl/well of FuGENE 6 transfection reagent (Promega, Madison, WI, United States). Expression of *Ano1* was confirmed by monitoring mKate2 fluorescence in cells.

HEK-293 cell lines expressing human Ca_*v*_3.2 channels were donated from Dr. E. Perez-Reyes (University of Virginia, VA). Generation of the cell lines and the electrophysiological properties of Ca_*v*_3.2 currents were described previously (Cribbs et al., 1998). Human Ca_*v*_3.2 (CACNA1H) was transfected into HEK 293 cells (A293; ATCC, Manassas, VA, United States) with vector (pcDNA3, Invitrogen). Cells were grown in 15 mM HEPES D-MEM/F-12 media (GIBCO, Carlsbad, CA, United States; with L-glutamine and pyridoxine hydrochloride) supplemented with 10% (v/v) fetal bovine serum (Gibco), 1% (v/v) pencillin-streptomycin (Gibco) at 37°C in 95% O_2_-5% CO_2_.

### Solutions and Drugs

Muscles used for Ca^2+^ imaging were perfused constantly with KRB solution containing (mmol/L): NaCl, 120.35; KCl, 5.9; NaHCO_3_, 15.5; NaH_2_PO_4_, 1.2; MgCl_2_, 1.2; CaCl_2_, 2.5; and glucose, 11.5. KRB solution was bubbled with a mixture of 97% O_2_ – 3% CO_2_ and warmed to 37 ± 0.2°C. Drugs used to inhibit CaCC were 5-nitro-2-(3-phenylpropylamino)-benzoic acid [NPPB; purchased from Sigma; (Yang et al., 2008)] and 2-[(5-Ethyl-1,6-dihydro-4-methyl-6-oxo-2-pyrimidinyl)thio]-*N*-[4-(4-methoxyphenyl)-2 thiazolyl]acetamide [T16Ainh-A01; purchased from Tocris Bioscience (Ellisville, MO, United States); (Namkung et al., 2011)]. Drugs used to inhibit NCX were 2-[2-[4-(4-Nitrobenzyloxy) phenyl]ethyl] isothiourea mesylate [KB-R7943; purchased from Tocris Bioscience; (Watano et al., 1996)] and 2-[[4-[(4-Nitrophenyl)methoxy]phenyl]methyl]-4-thiazolidinecarboxylic acid ethyl ester [SN-6; purchased from Tocris Bioscience; (Niu et al., 2007)]. NPPB (100 mM), T16Ainh-A01 (50 mM), KB-R7943 (10 mM) and SN-6 (10 mM) were dissolved in dimethyl sulphoxide (DMSO). Final dilutions were made by adding stock solutions to extracellular solutions used for specific experiments. Final concentrations of DMSO were less than 0.1%.

### Statistical Analyses

Data are expressed as means ± SEM of n cells for patch clamp experiments and *n* = numbers of tissues, each from a separate animal, in Ca^2+^ imaging experiments. All statistical analyses were performed using Graphpad Prism. We used Student’s *t*-test to compare single values under control and experimental conditions. A Mann–Whitney test was used for statistical comparisons of unpaired, non-parametric data sets (e.g., raw values of CTC duration). In all statistical analyses, *p* < 0.05 was considered statistically significant.

## Results

### Expression of Na^+^-Ca^2+^ Exchanger (Slc8a1-3) in Colonic and Small Intestinal ICC

**FIGURE 1:**
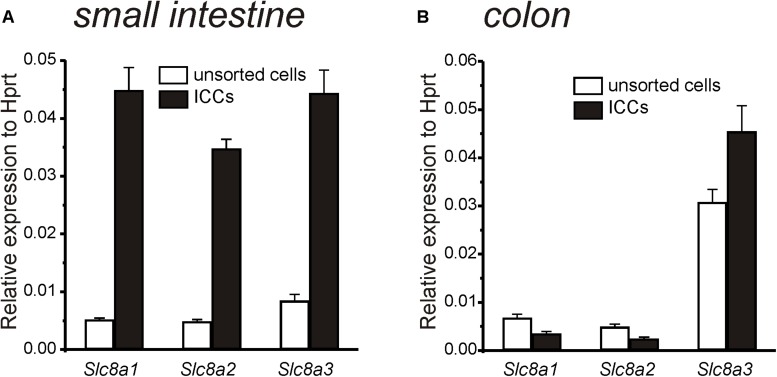
Quantitative analysis of Na^+^-Ca^2+^ exchanger (*Slc8a1-3*) expression in ICC and unsorted cells from small intestine and colon. **(A)** Summary graph showing relative expression of *Slc8a1, Slc8a2*, and *Slc8a3* in small intestinal ICC and in unsorted intestinal cells. Note greatly increased expression of all three isoforms in ICC of small intestine relative to the unsorted cells. **(B)** Summary graph showing relative expression of *Slc8a1, Slc8a2*, and *Slc8a3* in colon ICC and in unsorted colon cells. Only *Slc8a3* was elevated in colonic ICC. Expression of all transcripts was normalized to *Hprt*.

Transcripts of *Slc8a1, Slc8a2*, and *Slc8a3* were analyzed in ICC and enzymatically dispersed, but unsorted, cells from the *tunica muscularis* of the small intestine and colon. Previously we showed that ICC collected by FACS, as performed in this study, showed minimal expression of *Pdgfra* (a marker for fibroblast-like cells), *Myh11* (a marker for SMC) and *Uchl1* (a marker for neurons) and elevated levels of *Kit* transcripts (ICC marker) relative to the unsorted cells, suggesting that sorted ICC were highly purified by FACS (Peri et al., 2013). Compared to the unsorted, mixed cell population, *Slc8a1, Slc8a2* and *Slc8a3* were expressed at higher levels in small intestinal ICC, and *Slc8a3* was elevated in colonic ICC (Figures 1A,B).

### Ca^2+^ Exit Mode of NCX Is Favored Under Resting Conditions in ICC

**FIGURE 2:**
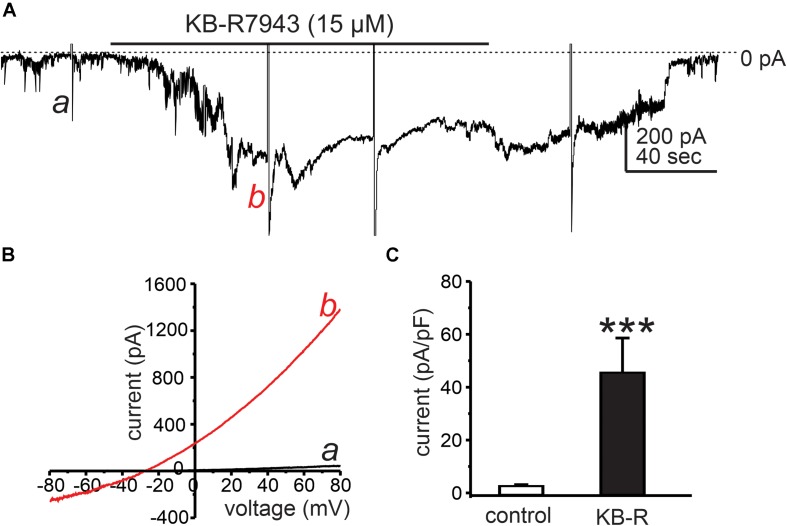
Cl^–^ conductance activated by KB-R7943. **(A)** Cells were dialyzed with internal solution (*E*_*Cl*_ = –40 mV, Solution VI in Table 2) and held at –50 mV. KB-R7943 (15 μM) activated inward current. Ramp potentials from –80 to +80 mV were applied before (example a in the trace) and after (example b in the trace) addition of KB-R7943 (15 μM). **(B)** Shows current responses to ramp potentials (a: controls, black trace in the panel) and b: KB-R7943 (15 μM), red trace in each panel). Data taken from traces in panel **A**. The currents activated by KB-R7943 (15 μM) were outwardly rectifying and reversed at *E*_*Cl*_. **(C)** Summarized data show average normalized currents (currents density, pA/pF) at 0 mV before (control) and after KB-R7943 (15 μM; *n* = 5). ***p < 0.001.

Under the basal conditions of our experiments, the reversal potential for NCX (*E*_*NCX*_) was estimated to be −0.4 mV using the equation *E*_*NCX*_ = 3*E*_*Na*_ – 2*E*_*Ca*_ at 30°C (Blaustein and Lederer, 1999). Thus, NCX would tend to function in the Ca^2+^ exit mode because resting membrane potentials and applied holding potentials under voltage clamp were negative to *E*_*NCX*_. Using Solution VI in patch pipettes (*E*_*Cl*_ = −40 mV; see Table 2) with external CaPSS solution (Solution I), KB-R7943 (15 μM), which is capable of blocking the Ca^2+^ exit mode of NCX (Watano et al., 1996) (IC_50_ = 32 μM; Hu et al., 2019) caused an increase in inward current at a holding potential of −80 mV (Figures 2A,B). Application of ramp potentials showed that the current that developed in response to KB-R7943, was outwardly rectifying (Figure 2B) and due to a Cl^–^ conductance because it reversed at *E*_*Cl*_ (i.e., current reversed at −29 ± 0.8 mV before correction for a 15 mV junction potential). KB-R7943 increased currents at 0 mV from 2.9 ± 0.9 to 29.9 ± 2.8 pA (*n* = 5, *p* < 0.01). Figure 2C shows averaged current density (pA/pF) at 0 mV before and after KB-R7943 (from 2.9 ± 0.2 pA/pF to 45.8 ± 12.8 pA/pF; *n* = 5).

### NCX and the Generation of Spontaneous Transient Inward Currents

**FIGURE 3:**
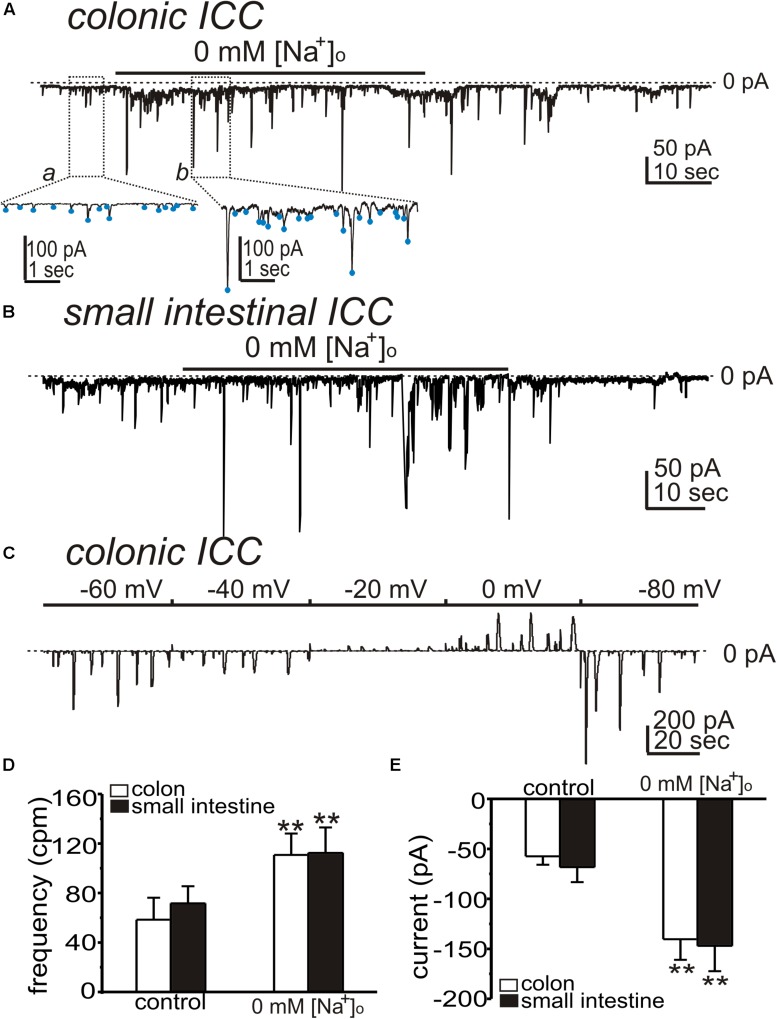
Effects of low [Na^+^]_o_ on spontaneous transient inward currents (STICs) in ICC. **(A,B)** Representative traces showing that replacement of [Na^+^]_o_ (140 mM) with equimolar Li^+^ (Solution II) increased the amplitude and frequency of STICs (holding potentials = –80 mV) in colonic **(A)** and small intestinal **(B)** ICC. Under control condition, the external solution was CaPSS (Solution I) and internal solution was Solution V (see Table 2). Insets show expanded time scale from upper panel (dotted boxes) to display how STIC amplitude and frequency were measured. Blue dots denote detection of STICs using event detection analysis (see section “Materials and Methods”). **(C)** Representative trace showing STICs at various potentials after 0 mM [Na^+^]_o_ in a colonic ICC. STICs reversed at about –29 mV (trace uncorrected for junction potential). Dotted lines denote 0 pA. **(D,E)** Summary data from five experiments shows the effects of low [Na^+^]_o_ on the frequency **(D)** and amplitude **(E)** of STICs (**p < 0.01).

Interstitial cells of Cajal generate STICs that are thought to be the basal pacemaker activity in ICC (Sanders et al., 2014). We tested the effects of shifting *E*_*NCX*_ to more negative potentials (to drive NCX into Ca^2+^ entry mode) on STICs by reducing extracellular Na^+^ ([Na^+^]_o_). Cells were held at −80 mV. After STICs were recorded under control conditions (i.e., external solution was Solution I with [Na^+^]_o_ = 140 mM and the pipette solution was Solution V), [Na^+^]_o_ was replaced with 140 mM Li^+^ (Solution II), which would be predicted to shift *E*_*NCX*_ to very negative potentials. Replacement of [Na^+^]_o_ increased the frequency and amplitude of STICs from 58 ± 18 cpm to 111 ± 17 cpm (*n* = 5, *p* < 0.01; Figures 3A,D) and from −58 ± 8 pA to −140 ± 21 pA (*n* = 5, *p* < 0.01; Figures 3A,E) in colonic ICC, respectively. Small intestinal ICC showed similar responses to replacing [Na^+^]_o_. The frequency and amplitude of STICs increased from 72 ± 14 cpm to 112 ± 21 cpm (*n* = 5, *p* < 0.01; Figures 3B,D) and from −68 ± 15 pA to −147 ± 25 pA (*n* = 5, *p* < 0.01; Figures 3B,E). To determine whether the STICs activated by low [Na^+^]_o_ were due to a Cl^–^ conductance, holding potentials were changed from −80 to 0 mV (Figure 3C, *n* = 5). STICs reversed at −28 ± 2 mV (*n* = 5) when cells were dialyzed with Solution VI (not corrected for 15 mV junction potential). These data suggest that STICs, activated by reducing [Na^+^]_*o*,_ were due to a Cl^–^ conductance and reversed at the calculated *E*_*Cl*_.

### Activation of Ca^2+^-Activated Cl^–^ Conductance by Ca^2+^ Entry Mode of NCX

**FIGURE 4:**
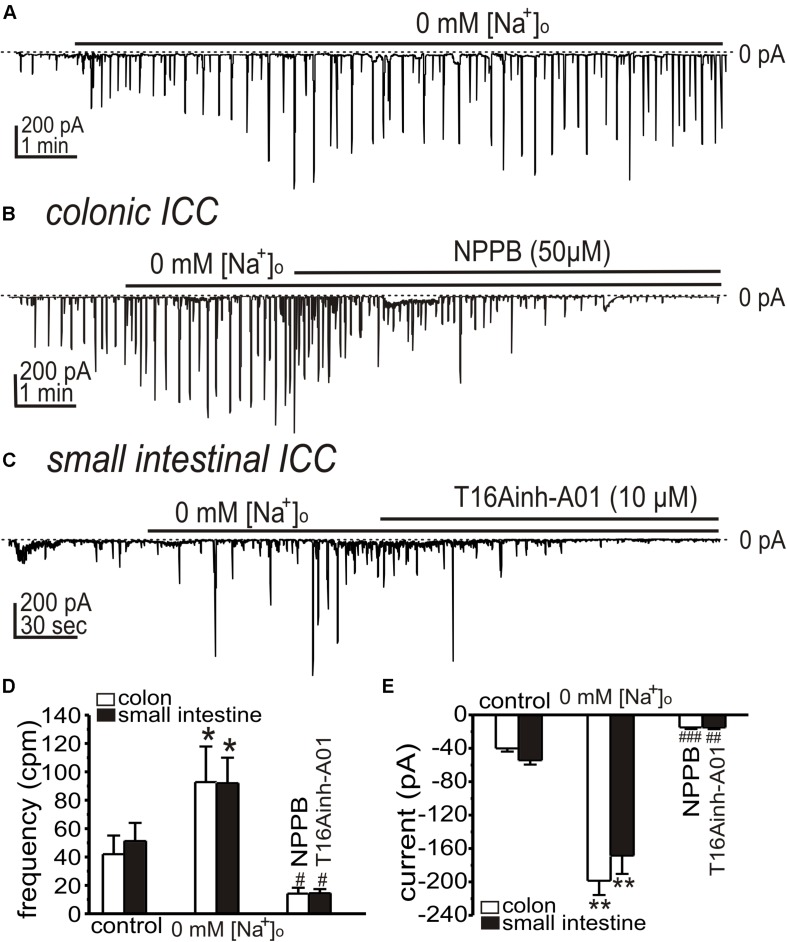
Effects of Ca^2+^-activated Cl^–^ channel (CaCC) blockers on STICs activated by low [Na^+^]_o_ in colonic and small intestinal ICC. **(A–C)** Representative traces showing increase in STICs in response to low external [Na^+^]_o_ (see Solution II). **(A)** Low external [Na^+^]_o_ increased STICs for the duration of the exposure (in this 10 min; small intestinal ICC). STICs were inhibited by CaCC blockers. **(B)** NPPB (50 μM) inhibited STICs in colonic ICC held at –80 mV (Internal Solution V). **(C)** STICs were inhibited in small intestinal ICC by T16Ainh-A01 (10 μM). Dotted lines denote 0 pA. **(D,E)** Summary data showing the effects of low [Na^+^]_o_ and Ano1 blockers on the frequency **(D)** and amplitude **(E)** of STICs (*p < 0.05, **p < 0.01 vs. control; #*p* < 0.05, ##*p* < 0.01, ###*p* < 0.001 vs. low [Na^+^]_o_).

In the Ca^2+^ entry mode of NCX, Ca^2+^ entering cells may increase [Ca^2+^]_*i*_ and activate CaCC. We have previously shown that *Ano1* transcripts and protein are highly expressed in ICC (Hwang et al., 2009; Zhu et al., 2009), and we tested inhibitors of these channels. First we tested whether the STICs activated by low [Na^+^]_o_ are sustained for periods required to test CaCC inhibitors. Replacing [Na^+^]_o_ (Solution II) increased STICs, as above, and the activation of STICs was sustained for at least 10 min (Figure 4A). NPPB (50 μM) inhibited the amplitude of STICs activated in colonic ICC from −199 ± 17 to −15 ± 2 pA (*n* = 5, *p* < 0.01; Figures 4B,E), respectively. T16Ainh-A01 (10 μM), a second and structurally different ANO1 antagonist, inhibited the STICs in small intestinal ICC from 92 ± 18 to 14 ± 3 cpm (*n* = 5, *p* < 0.05; Figures 4C,D) and from −168 ± 22 to −15 ± 2 pA (*n* = 5, *p* < 0.01; Figures 4C,E), respectively.

**FIGURE 5:**
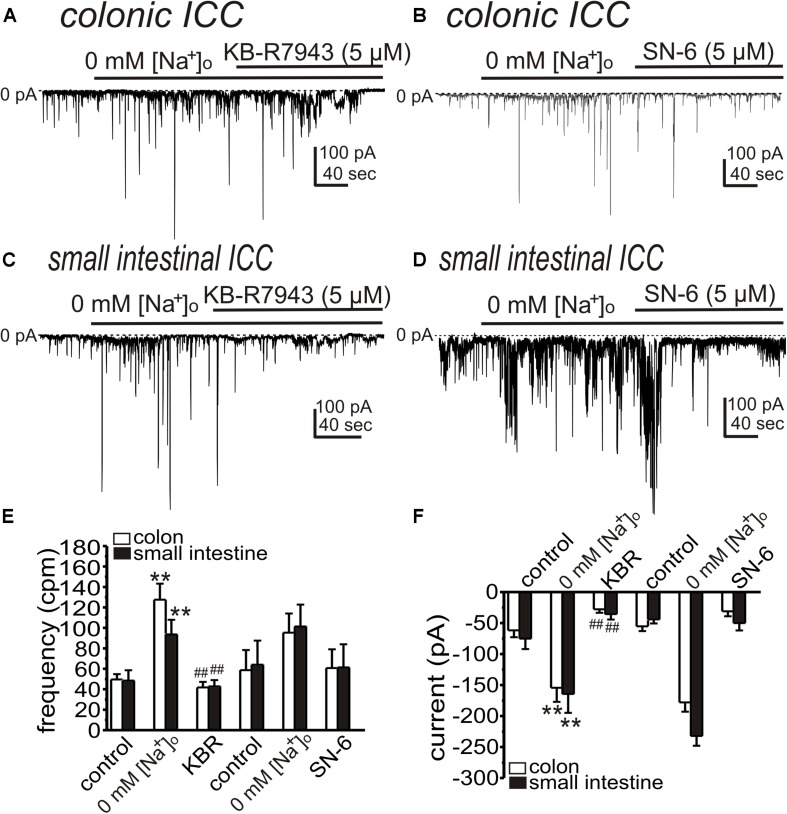
KB-R7943 and SN-6 inhibit STICs activated by low [Na^+^]_o_. Cells were held at –80 mV and STICs occurred spontaneously. **(A,B)** Representative traces showing that KB-R7943 (5 μM) and SN-6 (5 μM) inhibited STICs activated by 0 mM [Na^+^]_o_ (Solution II) in colonic ICC. **(C,D)** Representative traces showing that KB-R7943 (5 μM) and SN-6 (5 μM) inhibited STICs activated by 0 mM [Na^+^]_o_ in small intestinal ICC. Dotted lines denote 0 pA. **(E,F)** Summary data showing the effects of low [Na^+^]_o_ and Ano1 blockers on the frequency **(D)** and amplitude **(E)** of STICs. **p < 0.01 vs. control; ##*p* < 0.01 vs. low [Na^+^]_o_.

We also tested the effects of NCX blockers (KB-R7943 and SN-6) on STICs after reduced [Na^+^]_o_. KB-R7943 (5 μM) and SN-6 (5 μM) significantly reduced the amplitude of STICs in colonic ICC (from 128 ± 16 to 42 ± 5 cpm and from −154 ± 23 to −28 ± 6 pA (*n* = 5, Figures 5A,E,F) and from 95 ± 19 to 61 ± 18 cpm and from −178 ± 15 to −31 ± 8 pA [*n* = 3, Figures 5B,D,E)], respectively. KB-R7943 (5 μM) and SN-6 (5 μM) reduced STICs in small intestinal ICC (from 94 ± 14 to 43 ± 6 cpm and from −164 ± 30 to −36 ± 9 pA [*n* = 5, Figures 5C,E,F)] and from 101 ± 21 to 61 ± 23 cpm and from −232 ± 16 to −50 ± 12 pA (*n* = 3, Figures 5D,E,F), respectively).

### Ca^2+^ Entry Mode of NCX Contributes to the Time-Course of Slow Wave Currents

**FIGURE 6:**
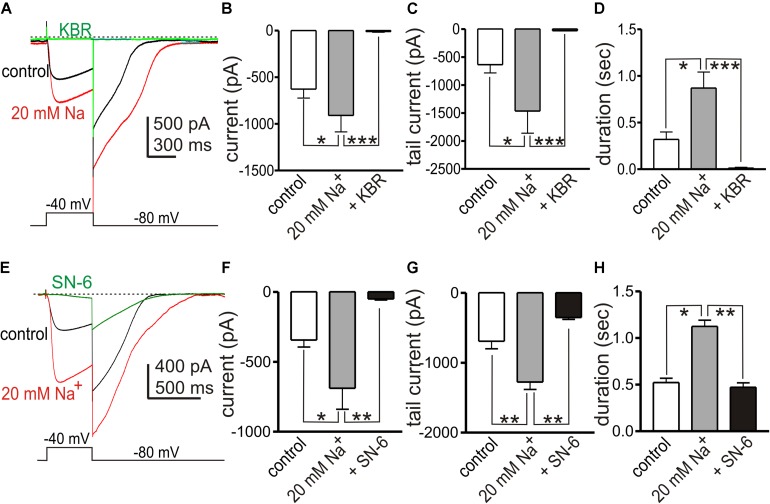
Effects of shifting *E*_*NCX*_ to more negative potentials on slow wave currents in small intestinal ICC. **(A,E)** Representative traces showing slow wave currents in small intestinal ICC evoked by step depolarizations (500 ms) from –80 to –40 mV. Repolarization to holding potential (–80 mV) resulted in long lasting tail currents that exceeded the deactivation properties of ANO1 (45). The sustained tail currents demonstrate the autonomous nature of the slow wave currents (i.e., once activated these responses persist even after repolarization). Reduced [Na^+^]_o_ (to 20 mM; 115 mM Na^+^ replaced with equimolar Li^+^, Solution III) increased the amplitude of slow wave currents and the amplitude and duration of the tail currents (red traces), as compared to control currents (with 140 mM [Na^+^]_o_, black traces). **(A)** KB-R7943 (5 μM, KBR) and **(E)** SN-6 (5 μM) reduced the slow wave currents and tail currents (green traces). **(B–D)** Summary data showing the effects of low [Na^+^]_o_ and KB-R7943 on the amplitude of slow wave currents **(B)**, the peak amplitude of tail currents **(C)** and the duration of tail currents **(D)** from 5 experiments. **(F–H)** Summary data showing the effects of low [Na^+^]_o_ and SN-6 on the amplitude of slow wave currents **(F)**, the peak amplitude of tail currents **(G)**, and the duration of tail currents **(H)** from four experiments. *p < 0.05, **p < 0.01, ***p < 0.001.

In previous studies we found that the conductance responsible for STICs in small intestinal ICC can be coordinated into whole-cell “slow wave currents” by step depolarizations and activation of low threshold, voltage-dependent Ca^2+^ channels (Zhu et al., 2009; Zheng et al., 2014). STICs are transient events averaging 334 ± 45 ms (*n* = 10) in small intestinal ICC, but slow wave currents display an “autonomous” nature and persist for at least 1.8 ± 0.2 s (*n* = 7), even after cells are repolarized (Goto et al., 2004; Zhu et al., 2009). We investigated whether the Ca^2+^ entry mode of NCX may be involved in the sustained time-course of ANO1 activation during slow wave currents. In whole cell configuration, slow wave currents were activated by step depolarization (0.5 s) from −80 to −40 mV using Solution I in the bath and Solution V as the pipette solution. Large amplitude tail currents, as shown with ANO1 channels expressed in HEK cells (Yu et al., 2014; Sung et al., 2016), persisted after repolarization to −80 mV, indicating sustained activation of ANO1 (Zhu et al., 2016). Na^+^ replacement with 115 mM Li^+^ (yielding 20 mM [Na^+^]_o_; Solution III) increased the amplitude of slow wave currents (from −627 ± 96 to −910 ± 176 pA, *n* = 5, *p* < 0.05), tail currents (from −636 ± 148 to −1466 ± 396 pA, *p* < 0.05) and the durations of tail currents (from 0.32 ± 0.07 s to 0.87 ± 0.17 s, *p* < 0.05, Figures 6B–D). KB-R7943 (5 μM) abolished the slow wave currents (Figures 6A–D, *n* = 5). SN-6 had similar effects on slow wave currents as KB-R7943. In experiments testing SN-6, the peak amplitude of slow wave current was −344 ± 50 pA in control (*n* = 4) and increased after addition of 20 mM [Na^+^]_o_ (Solution III) to −688 ± 150 pA (*p* < 0.05 vs. control). SN-6 (5 μM) decreased the peak amplitude of slow wave current to −51 ± 7 pA (*p* < 0.01 as compared to the amplitude with 20 mM [Na^+^]_o_) (Figures 6E,F). Tail current amplitude and duration were also decreased by SN-6 (Figures 6G,H).

### Effects of SN-6 and KB-R7943 on Key Slow Wave Current Components

**FIGURE 7:**
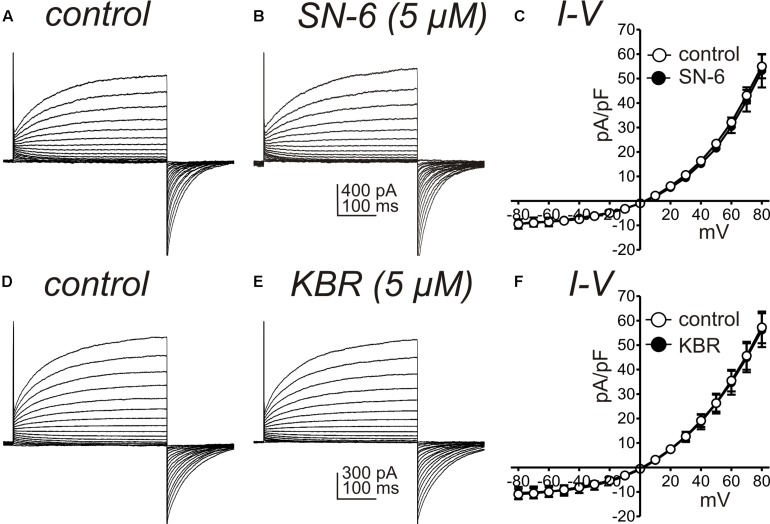
Effects of NCX antagonists on ANO1 currents expressed in HEK 293 cells. **(A,B)** show ANO1 current responses from HEK 293 cells transfected with the AC splice variant of *Ano1* to step protocols from –80 mV to +80 mV before and in the presence of SN-6 (5 μM). A symmetrical Cl^–^ gradient was established for these experiments (i.e., external and internal solutions were Solutions IV and VII, respectively). SN-6 had no effect on the ANO1 currents, and data from 5 cells are summarized in **(C)**. **(D,E)** Show ANO1 current responses from HEK 293 cells transfected with the AC splice variant of *Ano1* to step protocols from –80 mV to +80 mV before and in the presence of KB-R7943 (KBR; 5 μM). Peak currents at 400 ms during step depolarization were divided by cell capacitance and reported as current densities (pA/pF). KBR had no effect on the ANO1 currents, and data from 5 cells are summarized in **(F)**.

Another interpretation of the effects of SN-6 and KB-R7943 on STICs and slow wave currents could be a direct blocking effect of these drugs on the channels involved in these currents. Therefore, we performed control experiments to determine if KB-R7943 and SN-6 block ANO1 currents, which contribute to both STICs and slow wave currents. HEK 293 cells expressing ANO1 channels were stepped from −80 to +80 mV under whole cell voltage clamp conditions (Solution IV for bath solution and Solution VII for pipette solution). After obtaining control currents, the cells were exposed to KB-R7943 (5 μM) or SN-6 (5 μM) and the voltage clamp protocol was repeated. Neither of these agents had resolvable effects on ANO1 currents, as shown by the raw current traces (Figures 7A,B,D,E) or the I-V relationship (Figures 7C,F; *n* = 5 each).

**FIGURE 8:**
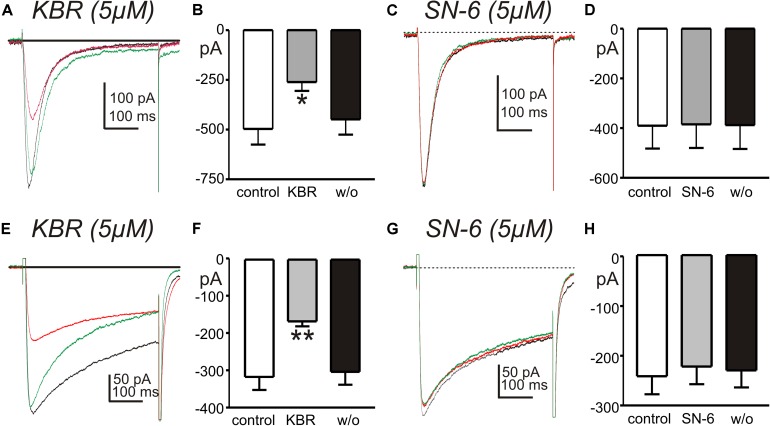
Effects of NCX antagonists on T- and L-type Ca^2+^ currents. **(A,C)** Show current responses to step depolarizations of HEK 394 cells (–80 to –40 mV) expressing Ca_*V*_3.2 channels before (black traces), in the presence of KB-R7943 (5 μM, KBR) or SN-6 (5 μM) (red traces), and after wash out of the drugs (green traces). **(B,D)** Summaries of the effects of KB-R7943 and SN-6 on peak T-type Ca^2+^ currents in 5 and 4 cells, respectively (*p < 0.05). **(E,G)** Show L-type Ca^2+^ currents evoked by step depolarizations of colonic SMC (–80 to 0 mV) before (black traces), in the presence of KB-R7943 (5 μM, KBR) or SN-6 (5 μM) (red traces), and after wash out of the drugs (green traces). **(F,H)** Summaries of the effects of KB-R7943 and SN-6 on peak L-type Ca^2+^ currents (*n* = 5 cells for each experiment; **p < 0.01).

Experiments were also performed to test the effects of KB-R7943 and SN-6 on T- and L-type Ca^2+^ currents, since these conductances are present in ICC and may help organize the openings of ANO1 channels into slow wave currents (Zheng et al., 2014). Ca_*V*_3.2 currents were recorded in whole cell voltage-clamp experiments from HEK 293 cells transfected with CACNA1H. Cells were exposed to CaPSS (Solution I) and dialyzed with Cs^+^-rich solution (Solution V) and stepped repeatedly from −80 to −40 mV. KB-R7943 (5 μM) decreased the amplitude of the inward current evoked by depolarization from −496 ± 79 to −261 ± 44 pA (*n* = 5, *p* < 0.05, Figures 8A,B). However, SN-6 (5 μM) had no significant effect on the amplitude of the Ca_*V*_3.2 currents (Figures 8C,D). The effects of KB-R7943 and SN-6 were also tested on L-type Ca^2+^-currents, using colonic SMC that express these channels (Koh et al., 2001). Cells were permeabilized with amphotericin to obtain whole-cell configuration. Inward currents were evoked by repeated step depolarization from −80 to 0 mV (Figure 8E). KB-R7943 (5 μM) significantly decreased the peak amplitude of L-type Ca^2+^ currents from −314 ± 35 pA to −166 ± 29 pA (*n* = 5, *p* < 0.01, Figures 8E,F). In contrast, SN-6 (5 μM) had no effect on L-type Ca^2+^ currents evoked at 0 mV (*n* = 5, Figures 8G,H). These data suggest that the effects of KB-R7943 on slow wave currents (Figures 6A–D) could have been contaminated by off target effects on T-currents. This might have caused the complete block of the slow wave currents as noted in Figure 6. In contrast SN-6 did not have display these cross-contaminating effects.

### Role of NCX in Maintenance of Ca^2+^ Transients

**FIGURE 9:**
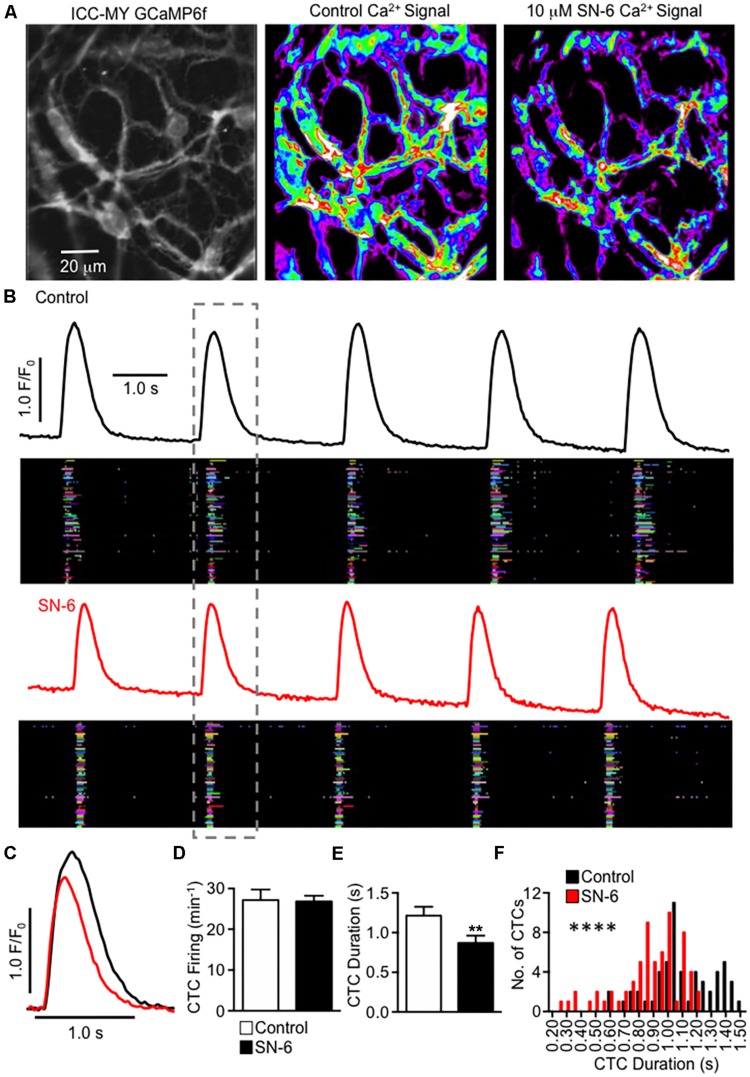
Inhibiting NCX decreases the duration of Ca^2+^ transient clusters in ICC-MY networks. **(A)** Raw image of an ICC-MY network recorded from the jejunum of a Kit-Cre-GCaMP6f mouse (far left panel). The summated heat map of all Ca^2+^ transients that occurred in this field of view (FOV) over a 30 s recording is shown before and after addition of SN-6 (10 μM) in the middle and far right panel, respectively. **(B)** Traces of summated Ca^2+^ activity in the ICC-MY network before (black) and after (red) addition of SN-6 (10 μM). Occurrence maps show the activity of each individual firing site within the ICC-MY network plotted against time, with each firing site in the network plotted as a differently colored lane. The number of colored lanes (counted along the *y*-axis) indicates the number of active firing sites. The width of each lane (measured along the *x* axis) at any point indicates the duration that the site was active. Note that under control conditions, many sites fired multiple Ca^2+^ transients during each cycle, but after SN-6, the number of occurrences at each site decreased to only a single event. **(C)** Expanded timescale showing a single CTC highlighted in the dashed gray box in **(B)**, before (black) and after (red) addition of SN-6 (10 μM). **(D)** Summary effect of SN-6 (10 μM) on CTC firing frequency, *n* = 6. **(E)** Summary effect of SN-6 (10 μM) on CTC duration, *n* = 6. **p < 0.01. **(F)** Histogram showing the distribution of values for CTC duration before (black) and after (red) addition of SN-6 (10 μM; *n* = 6). ****p < 0.0001.

Pacemaker activity in the small intestine arises from Ca^2+^ release events that activate CaCC in ICC within the plane of the myenteric plexus (ICC-MY) (Drumm et al., 2017). Ca^2+^ signaling in the ICC network occurs as temporally clustered firing from multiple Ca^2+^ release sites (Ca^2+^ transient clusters, CTCs (Drumm et al., 2017). We imaged CTCs in intact ICC-MY networks *in situ* using Kit-Cre-GCaMP6f mice (see Methods). Under control conditions, ICC-MY firing rhythmic CTCs at a frequency of 27.2 ± 2.6 min^–1^ (Figures 9A,B, *n* = 6). SN-6 (10 μM) did not change the frequency of CTCs (Figures 9B–D, 26.8 ± 1.4 min^–1^, *P* = 0.87, *n* = 6), but the heat maps of CTC activity indicated that the total CTC Ca^2+^ signal was decreased by SN-6 (Figure 9A). Occurrence maps, plotting Ca^2+^ transients originating from individual firing sites in ICC-MY, revealed that firing sites were not as active in the presence of SN-6 (Figure 9B). Close examination showed that the duration of CTCs was decreased by SN-6. A single CTC is highlighted in Figure 9B and expanded in Figure 9C to show that when the onset of CTC firing was overlapped there was a significant reduction in CTC duration in the presence of SN-6 (Figure 9C). The average duration of CTCs was 1.2 ± 0.1 s under control imaging and this was reduced to 0.87 ± 0.09 s by SN-6 (Figure 9E, *n* = 6). The raw values of CTC duration were exampled and plotted as a frequency histogram (Figure 9F). The histogram demonstrates that SN-6 caused a leftward shift in the distribution of CTC durations, and this was statistically significant using a Mann–Whitney test (Figure 9F, *P* < 0.0001, *n* = 6).

### The Influence of Na^+^ Pump on the Amplitude and Duration of Slow Wave Currents

**FIGURE 10:**
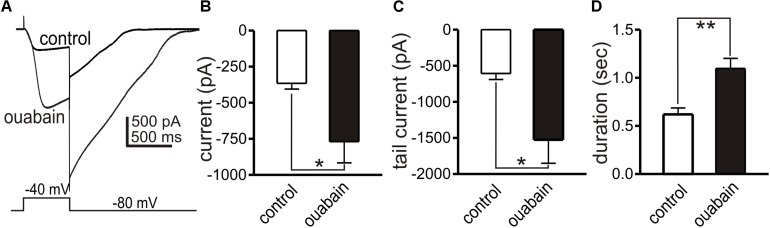
Effects of ouabain on slow wave currents. Slow wave currents, activated by depolarizing steps from –80 to –40 mV, are shown in **(A)** before and in the presence of ouabain (200 nM). Ouabain increased the amplitude of the slow wave current, the amplitude of the tail current upon repolarization to –80 mV and the duration of the tail current. Summarized data from five experiments are shown in **(B–D)**, respectively. *p < 0.05, **p < 0.01.

Modeling has suggested that a shift in the Na^+^ gradient, due to Na^+^ entry from the action of the Na^+^K^+^Cl^–^ cotransporter (NKCC1), may facilitate slow wave currents due to the Ca^2+^ entry mode of NCX (Youm et al., 2019). Restoration of the Na^+^ gradient is likely to occur via the actions of Na^+^K^+^ ATPase (Na^+^ pump). A gene array study performed on ICC found an average 6.5 fold higher expression of *Atp1a2* in ICC-MY in the small intestine vs. whole tissue (Chen et al., 2007). *Atp1a2* encodes the “α2” isozyme of the Na^+^ pump that is relatively sensitive to block by ouabain with a K_*d*_ of 27 nM (O’Brien et al., 1994). Therefore, we also tested the effects of ouabain on slow wave currents, using a concentration that would be relatively selective for the α2 isozyme. In whole cell configuration, slow wave currents were activated by step depolarization (0.5 s) from −80 to −40 mV. Ouabain (200 nM) increased the amplitude of slow wave currents (from −366 ± 40 to −769 ± 147 pA, *n* = 5, *p* < 0.05), tail currents after stepping back to −80 mV (from −607 ± 83 to −1525 ± 327 pA, *p* < 0.05), and durations of tail currents (from 0.6 ± 0.06 s to 1.1 ± 0.1 s, *p* < 0.01). Data from these experiments are summarized in Figure 10.

### Protein-Protein Interaction Between ANO1 and NCX3

**FIGURE 11:**
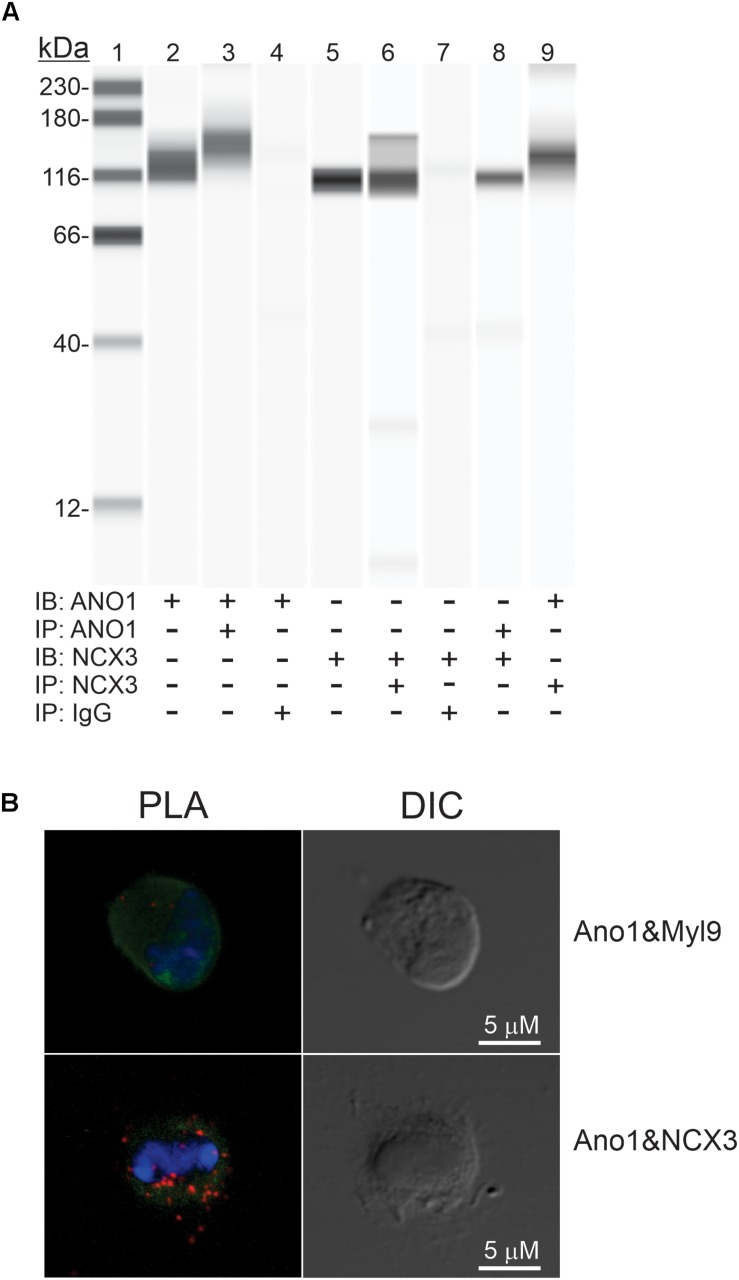
Protein-protein interaction between ANO1 and NCX3. **(A)** Co- IP of ANO1 and NCX3 from small intestine smooth muscle membrane fraction. Immunoprecipitates (IP) were obtained by elution with low pH buffer, and immunoblotting (IB) was performed using Wes with anti-ANO1 and anti-NCX3 antibodies. Lanes: 1, protein standards; 2, ANO1 in membrane fraction (2.5 mg); 3, ANO1 in ANO1 immunoprecipitate (5 ml); 4, ANO1 in non-immune rabbit IgG IP; 5, NCX3 in membrane fraction (1 mg); 6, NCX3 in NCX3 immunoprecipitate (5 ml); 7, NCX3 in non-immune rabbit IgG IP; 8, NCX3 in Ano1 immunoprecipitate (5 ml); 9, ANO1 in NCX3 immunoprecipitate (5 ml) *n* = 4. **(B)** Association between ANO1 and NCX3 occurs *in situ* in ICC, as demonstrated by PLA. ICC from small intestine (green, left panel) were exposed to the rabbit anti-ANO1 and goat anti-NCX3 antibodies, followed by the Duolink minus-anti rabbit IgG and plus-anti goat IgG secondary antibodies and the ligation and amplification reactions. For the PLA negative control, ICC were exposed to rabbit anti-ANO1 and mouse anti-myosin regulatory light chain antibodies (upper left panel). PLA-positive red spots were detected by ANO1-NCX3 co-localization (lower left panel). Few red spots were observed in negative controls. Nuclear staining of ICC was achieved with DAPI (blue, left panel). Intact ICC were confirmed by differential interference contrast (DIC) (right panels).

Co-immunoprecipitation assays, followed by Wes analysis of the immunoprecipitates, were utilized to investigate possible interactions between ANO1 and NCX3 proteins. Figure 11A, Lanes 2, and 5 show the presence of ANO1 (∼116 kDa) and NCX3 (∼100 kDa), respectively, in the input samples (small intestinal muscle membrane fraction) used for immunoprecipitation. Positive controls show that ANO1 (∼116 kDa) was detected by anti-ANO1 immunoblotting of the anti-ANO1 immunoprecipitate, and NCX3 (∼100 kDa) was detected by anti-NCX3 immunoblotting of the anti-NCX3 immunoprecipitate (Figure 11A, Lanes 3, 6, respectively). Negative controls show that in samples immunoprecipitated with non-immune rabbit IgG, both NCX3 and ANO1 were not detected by anti-NCX3 or anti-ANO1 immunoblotting of the non-immune rabbit IgG immunoprecipitate (Figure 11A, Lanes 4, 7, respectively). However, in samples immunoprecipitated with either the anti-ANO1 or anti-NCX3 antibodies, NCX3 (∼100 kDa) was detected by anti-NCX3 immunoblotting of the anti-ANO1 immunoprecipitate, and ANO1 (∼116 kDa) was detected by anti-ANO1 immunoblotting of the anti-NCX3 immunoprecipitate (Figure 11A, Lanes 8, 9, respectively), indicating a complex comprised of these two proteins.

The proximity of ANO1 and NCX3 in small intestine ICC *in situ* was investigated by PLA. ICC were exposed to ANO1 and NCX3 antibodies, each from a different host species, followed by the appropriate secondary antibodies each linked to + and – complementary oligos to capture the fluorophore probe (see section “Materials and Methods”). In the negative control, the PLA procedure was carried out on ICC exposed to anti-ANO1 and a myosin regulatory light chain antibody; two proteins expressed in ICC but located in distinct subcellular compartments (Lee et al., 2017). As shown in Figure 11B, several PLA-positive red spots were detected with the ANO1 and NCX3 antibodies, but few red spots were observed in the negative control. The normalized intensities of the PLA signal were 1.43 ± 0.15 (ANO1 + NCX3) and 0.14 ± 0.03 (ANO1 + myosin regulatory light chain, negative control), respectively (from 6 cells; *p* < 0.001).

## Discussion

This study supports a role for NCX in pacemaker activity of ICC from the murine intestine. Previously we and others have addressed the role of Ca^2+^ entry and Ca^2+^ release in the initiation of pacemaker activity in ICC (van Helden et al., 2000; Hirst et al., 2002; van Helden and Imtiaz, 2003; Park et al., 2006; Drumm et al., 2017), however, much about the dynamics and sources of Ca^2+^ in ICC is not yet understood. Modeling of slow waves (Youm et al., 2019) and empirical data obtained in the current study suggest that NCX switches from Ca^2+^ exit mode to Ca^2+^ entry mode during the slow wave cycle. The Ca^2+^ exit mode clears Ca^2+^ from the proximity of ANO1 channels maintaining a low open probability for these channels during the inter-slow wave interval. The Ca^2+^ entry mode contributes to the sustained activation of CaCC channels, creating the plateau phase of slow waves. The plateau phase of slow waves is a critical determinant of GI motility because depolarization during this phase initiates action potentials (as in the small intestine and colon) or results in sustained Ca^2+^ entry (as in the stomach) via activation of L-type Ca^2+^ channels in SMCs (Ozaki et al., 1991). *Slc8a1-3* genes are expressed in ICC of the small and large intestines, however, different profile of NCX isoform expression were observed in ICC of the two regions. The implications of differences in NCX gene expression are not understood at the present time. Evidence also suggests that protein-protein interactions occur between NCX3 and ANO1 proteins and these proteins are closely associated in ICC *in situ*. The close proximity between these proteins may increase the efficiency of NCX3 in removing and delivering Ca^2+^ to ANO1.

Previous studies have indicated that the basic pacemaker activity in ICC is the generation of STICs, and these events produce STDs (also called unitary potentials by some authors) during membrane potential recording (current clamp conditions) (Edwards et al., 1999; van Helden et al., 2000, 2010; Zhu et al., 2011). STICs and STDs result from Ca^2+^ release from ER [e.g., STICs are reduced by inhibition of IP_3_ and ryanodine receptors (Zhu et al., 2015)] and activation of CaCC, encoded by *Ano1* (van Helden et al., 2000; Zhu et al., 2011). Our results suggest that ongoing Ca^2+^ exit mode of NCX serves to maintain low Ca^2+^ near CaCC because treatment with the NCX inhibitor, KB-R7943, activated sustained CaCC (Figure 2). Due to the close association of NCX and ANO1, the increase in [Ca^2+^]_*i*_ due to inhibition of the Ca^2+^ exit mode may be restricted to microdomains where Ca^2+^ release activates ANO1 channels. Shifting the reversal potential of NCX to negative potentials by replacement of extracellular Na^+^ with Li^+^ appears to flip NCX from Ca^2+^ exit mode to Ca^2+^ entry mode. This change increased the amplitude and frequency of STICs. This finding suggests that Ca^2+^ entry via NCX regulates the excitability of ER Ca^2+^ release channels. Increasing STICs would have the effect of causing depolarization in intact muscles with the increased inward currents summing in thousands of ICC.

Potential non-specific effects of the NCX blockers were tested in a series of control experiments. Neither KB-R7943 nor SN-6 directly affected ANO1 current, the conductance responsible for STICs and slow wave currents. STICs and slow wave current rely upon release of Ca^2+^ from stores, and both events are inhibited by drugs that block ER Ca^2+^ channels (IP_3_ receptor-operated and ryanodine receptors). Reduced extracellular Na^+^, a maneuver to shift the reversal potential for NCX, enhanced STICs. KB-R7943 and SN-6 reduced the STICs enhanced by low extracellular Na^+^, but did not block these events. This observation suggests that KB-R7943 and SN-6 did not block Ca^2+^ release from stores. Voltage-dependent Ca^2+^ currents (via T-channels) coordinate Ca^2+^ release from stores in ICC and thus control the openings of ANO1 channels that are responsible for slow wave currents (Zhu et al., 2009; Sanders et al., 2014; Zheng et al., 2014). Thus, block of T-type Ca^2+^ currents is expected to block slow wave currents. Such an observation was reported previously, as Ni^2+^ (30 μM) was shown to inhibit slow wave currents in ICC (Zhu et al., 2009). We found that KB-R7943 partially inhibited L- and T-type (Ca_*V*_3.2) currents (Figure 8). Thus, it is possible that the effects of this compound on slow wave currents could have been mediated partially by suppression of Ca^2+^ entry via voltage-dependent Ca^2+^ channels. There is no known dependence of STICs upon L- or T-type Ca^2+^ channels, so the action of KB-R7943 and SN-6 were likely to be free from contamination in reducing STICs. SN-6 had no effects on ANO1 currents nor on L- and T-type currents at the concentration used to inhibit NCX, and this compound significantly reduced STICs and slow wave currents in isolated ICC and the duration of CTCs in ICC-MY *in situ*.

Immunohistochemistry studies have demonstrated specific expression of ANO1 protein in ICC (Gomez-Pinilla et al., 2009; Hwang et al., 2009). Our data showed that protein-protein interactions occur between ANO1 and NCX3 in small intestinal muscles. It is also apparent that NCX3 is in close proximity to ANO1 channels in ICC *in situ*, as demonstrated by PLA. Close association between NCX3 and ANO1 may increase the efficiency of NCX in removing Ca^2+^ from microdomains near ANO1 channels during slow wave repolarization and during the inter-slow wave interval.

Based on the ionic conditions of our experiments and assuming low initial Na^+^ concentration and Ca^2+^ concentrations in microdomains between ER and plasma membranes, we calculated *E*_*NCX*_ to be in the range of −0.4 mV (at 30°C), thus favoring the Ca^2+^ exit mode at the resting potentials of ICC. Shifting *E*_*NCX*_ to negative potentials by reducing extracellular Na^+^ increased STICs at negative holding potentials. This observation suggests that NCX can flip to the Ca^2+^ entry mode and link to Ca^2+^-induced Ca^2+^ release to enhance STICs.

STICs occur in a stochastic manner in ICC, but CaCC openings synchronize periodically to develop slow wave currents (Goto et al., 2004; Zhu et al., 2009). Slow wave currents, sustained in the ICC of some GI muscles for several secs, behave “autonomously” after activation by depolarization (Goto et al., 2004; Zhu et al., 2009). How ANO1 remains activated, which is dependent upon Ca^2+^ remaining elevated, during the plateau phase of slow waves has not been determined. Our data suggest that the longevity of the plateau phase is related to the duration of time that NCX remains in Ca^2+^ entry mode. Shifting *E*_*NCX*_ of NCX to more negative potentials and creating a greater driving force for Ca^2+^ entry mode increased the duration of slow wave currents, and an inhibitor of NCX (SN-6) reduced the duration of slow wave currents and CTCs in ICC-MY *in situ*. When NCX switches to Ca^2+^ entry mode, the close association between NCX3 and ANO1 may be important for delivering Ca^2+^ to Ca^2+^ release channels in the ER to initiate Ca^2+^ transients and activation of ANO1 channels in the plasma membrane.

How NCX might flip to Ca^2+^ entry mode during the course of the slow wave cycle is of interest. Activation of CaCC during slow wave currents causes Cl^–^ efflux. ANO1 channels appear to be localized in microdomains in ICC (Zhu et al., 2015). Slow wave currents measured from single ICC reach 1 nA in amplitude and average 147 pA/pF in current density (Zhu et al., 2009). Efflux of Cl^–^ would decrease during the plateau phase of slow waves because the ICC depolarize close to *E*_*Cl*_. To maintain Cl^–^ homeostasis, Cl^–^ recovery must occur, and previous studies and modeling have suggested a role for the Na-K-Cl co-transporter (NKCC1) in this process (Wouters et al., 2006; Zhu et al., 2016; Youm et al., 2019). NKCC1 utilizes energy from the Na^+^ gradient to concentrate Cl^–^ ions in cells (Russell, 2000) and recover Cl^–^ lost during slow waves. Cl^–^ recovery by NKCC1 results in Na^+^ influx equal to half the molar amount of Cl^–^ lost through Cl^–^ current. We hypothesize that Cl^–^ recovery during the plateau phase of slow waves increases [Na^+^]_*i*_ in the restricted volumes of microdomains. We can also estimate that [Ca^2+^] in the vicinity of CaCC must rise to at least 1 μM because the outward rectification of CaCC is lost during slow wave currents (Zhu et al., 2009). If, at the peak of slow waves, [Ca^2+^]_*i*_ rises to 1 μM and [Na^+^]_*i*_ rises to 15–20 mM, *E*_*NCX*_ would shift negative to the potential reached during the plateau phase in ICC (about −10 mV or *E*_*Cl*_) (Kito and Suzuki, 2003; Kito et al., 2005). Thus, conditions favorable to Ca^2+^ entry mode would be achieved during the plateau phase. Influx of Na^+^ would decrease as [Cl^–^]_*i*_ is restored, and resting [Na^+^]_*i*_ would be restored by the actions of the Na^+^ pump. As [Na^+^]_*i*_ declines, NCX would flip back into Ca^2+^ exit mode, [Ca^2+^]_*i*_ would be restored to resting levels, CaCC would deactivate, and ICC would repolarize to complete the slow wave cycle. Consistent with this idea, we found that ouabain, at concentrations likely to be specific for the α2 isoform of the Na^+^ pump, lengthened the duration of slow wave currents. For such a mechanism to function, as predicted by modeling (Youm et al., 2019), the changes in ion concentrations hypothesized would need to occur in restricted volumes (i.e., microdomains) within ICC because it is unlikely that slow wave currents would result in appreciable reduction in cytoplasmic [Cl^–^] or significant increases in cytoplasmic [Na^+^].

The α2 isoform of the Na^+^ pump has been localized in microdomains formed by junctions between the plasma membrane and the sarcoplasmic reticulum in cardiac and SMC (Luther et al., 1992; Juhaszova and Blaustein, 1997a, b; Despa and Bers, 2007; Linde et al., 2012). NCX exchangers have also been localized in these microdomains (Juhaszova and Blaustein, 1997a; Lynch et al., 2008; Davis et al., 2009), suggesting tight coordination between [Na^+^]_*i*_ and Ca^2+^ signaling in microdomains. Such an arrangement between α2 Na^+^ pump proteins and NCX is compatible with the findings of the present study. The close association between ANO1 and NCX suggest that a signaling complex, involving multiple molecular entities (i.e., a pacemaker some) is fundamental to the pacemaker activity of ICC.

A role for NCX in pacemaker activity has been suggested in studies of interstitial cells of the urethra (Bradley et al., 2006; Drumm et al., 2015) and small intestine (Lowie et al., 2011), however, this hypothesis was not tested on ICC under conditions in which *E*_*NCX*_ can be manipulated in single cells. In murine small intestine KB-R7943 reduced the amplitude and frequency of Ca^2+^ transients in networks of ICC in intact muscles. These authors suggested that NCX, working in Ca^2+^ entry mode, was responsible for Ca^2+^ store reloading. However, as shown by our control studies of the effects of KB-R7943 on voltage-dependent Ca^2+^ conductances, the reduction in slow waves by this compound could have been due to effects on voltage-dependent Ca^2+^ entry, an essential step in generation of slow waves (Drumm et al., 2017). In urethra interstitial cells, forcing *E*_*NCX*_ to more negative potentials by reducing extracellular [Na^+^] increased the frequency and amplitude of Ca^2+^ waves and STICs (Bradley et al., 2006; Drumm et al., 2015), consistent with our observations in ICC of the small intestine. However, these authors estimated resting *E*_*NCX*_ to be quite negative (−72 mV), and no obvious role was identified for the Ca^2+^ exit mode of NCX in urethral interstitial cells. Inhibiting NCX under basal conditions inhibited STICs and Ca^2+^ transients suggesting these events are supported by Ca^2+^ influx due to the Ca^2+^ entry mode NCX. It should be noted that when membrane potential was clamped positive and negative to the estimated *E*_*NCX*_, there was no significant difference in STIC generation. This suggests that either the *E*_*NCX*_ estimates were incorrect or that NCX, operating in Ca^2+^ entry mode is not essential for either generation of STICs (e.g., via Ca^2+^ induced Ca^2+^ release) or maintenance of STICs by store refilling. Our observations suggest a dynamic nature for NCX in the slow wave cycle in which Ca^2+^ exit mode helps to maintain basal [Ca^2+^]_*i*_ between slow waves and deactivate ANO1 at the end of the slow wave plateau, and Ca^2+^ entry mode sustains activation of ANO1 channels during the plateau phase of slow waves.

**FIGURE 12:**
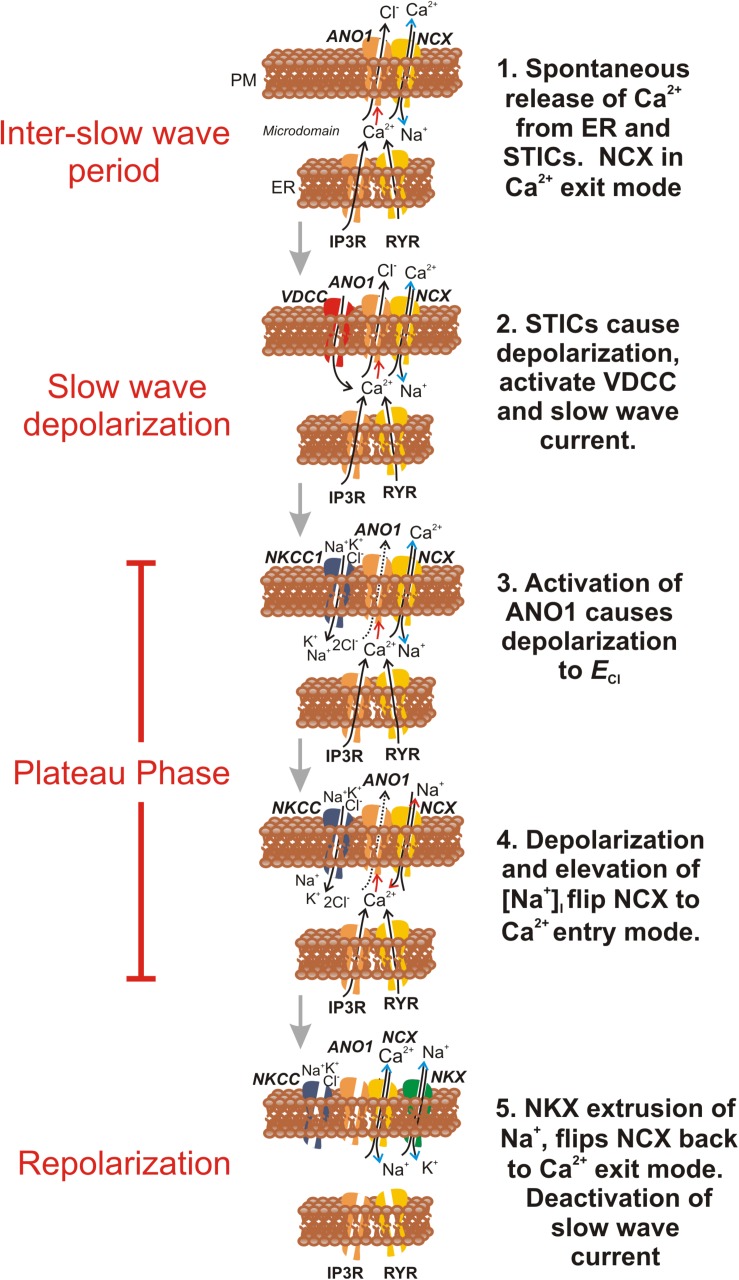
Schematic proposing how NCX contributes to ionic currents and fluxes during the slow wave cycle in ICC. This figure summarizes how the activity of NCX might be integrated with other known ionic mechanisms involved in generation of pacemaker activity in ICC. Electronmicroscopy has demonstrated close apposition between the plasma membrane and ER, and the restricted volumes formed are termed microdomains. Each frame in the schematic is a temporal representation of a typical microdomain, and ionic concentrations in the restricted volumes of the pacemaker units are hypothesized from current and previous findings. For simplicity, ion channels and transporters appear or disappear in individual frames as their role waxes and wanes. **Frame 1** shows the period of the inter-slow wave interval. Stochastic Ca^2+^ release events from the ER trigger (Red arrow) activation of ANO1 channels in the plasma membrane (PM), activating STICs. Following a Ca^2+^ transient, NCX, operating in Ca^2+^ exit mode (Blue arrow heads), and SERCA (not shown) rapidly restore basal [Ca^2+^]_*i*_ within the microdomain, reducing open probability of ANO1 channels and limiting the duration of STICs. **Frame 2** During the interval between slow waves, the probability of STICs increases with time (Hirst and Edwards, 2001). Summation of STDs (voltage response to STICs) causes activation of voltage-dependent (T-type) Ca^2+^ current (VDCC) (Zheng et al., 2014). Ca^2+^ entry triggers Ca^2+^-induced Ca^2+^ release in many microdomains in synchrony and activation of whole cell CaCC currents, known as the slow wave current (Zhu et al., 2009). Ca^2+^ entry and Cl^–^ efflux cause depolarization close to *E*_*Cl*_ (Kito et al., 2005). In **Frame 3** Cl^–^ efflux slows as membrane potential approaches *E*_*Cl*_, but Cl^–^ conductance remains high, clamping membrane potential for a second or more (plateau phase of slow wave) near *E*_*Cl*_. Cl^–^ loss is recovered by the action of the Na^+^K^+^2Cl^–^ (NKCC1) cotransporter (Wouters et al., 2006). This transporter restores [Cl^–^]_*i*_ in the microdomain using the energy of the Na^+^ gradient. In **Frame 4** accumulation of Na^+^ in the restricted volume of the microdomain flips NCX into Ca^2+^ entry mode (Red arrow heads), and the close association between Ca^2+^ entry through NCX, ANO1 channels and Ca^2+^ release channels (IP3R and RYR) helps to sustain Ca^2+^ transients and activation of ANO1, creating the sustained depolarization of the plateau phase. In **Frame 5** [Cl^–^]_*i*_ is restored and the accumulated Na^+^ is eventually removed by the combined actions of NCX and the Na^+^/K^+^ ATPase (NKX). Reducing [Na^+^]_*i*_ flips NCX back into the Ca^2+^ exit mode, and causes reduced [Ca^2+^]_*i*_ in the microdomain, deactivation of ANO1 and repolarization. Reloading of ER Ca^2+^ stores (by SOCE, Zheng et al., 2018; not shown) increases the probability of STICs and recharges the next slow wave cycle.

A final important point to note is that very little is known about the actual concentrations of ions in microdomains formed by close associations of ER and the plasma membrane in ICC. We have referred to these microdomains as “pacemaker units” previously, and they serve as an important structural feature of ICC (Sanders et al., 2014). Dynamic changes in [Ca^2+^], [Na^+^], [Cl^–^], Cl^–^ channels, Ca^2+^ release channels, NCX, NKCC1, Na^+^/K^+^ ATPase, and other transporters important for pacemaker activity and the development of slow wave currents may be focalized in microdomains, and relatively small ion fluxes may lead to significant concentration changes in these microdomains, as modeling has predicted (Youm et al., 2019). Direct measurement of the ionic concentrations in the restricted volumes of microdomains has not been accomplished. Although cells in our study were dialyzed with low [Na^+^]_*i*_ solutions, it is possible that the concentrations of ions in the pipette solutions did not accurately reflect the resting concentrations and changes in concentration occurring in microdomains during STICs and slow wave currents. Modeling has supported the hypothesis that T-type Ca^2+^ channels, ANO1 channels and NCX participate in the slow waves cycle, as depicted in Figure 12, and suggested that flipping NCX from Ca^2+^ exit to Ca^2+^ entry mode depends upon oscillations of [Na^+^] in microdomains in the mM range (Youm et al., 2019).

In summary, our results suggest that the bidirectional nature of NCX is exploited during the slow wave cycle in ICC to maintain low levels of [Ca^2+^]_*i*_ near ANO1 channels via Ca^2+^ exit mode during the inter-slow wave period. However, during slow waves, conditions occur (entry of Na^+^ and depolarization) that flip NCX into Ca^2+^ entry mode. Ca^2+^ entry facilitates Ca^2+^-induced Ca^2+^ release, the sustained occurrence of Ca^2+^ transients (i.e., CTCs), and sustains activation of ANO1, creating the plateau phase. In this study we have demonstrated a mechanism to explain how NCX might transition from Ca^2+^ exit to Ca^2+^ entry mode, and then how NCX might flip back to Ca^2+^ exit mode as a means of terminating slow wave currents and initiating slow wave repolarization. We have also observed a novel protein-protein interaction between NCX3 and ANO1 channels that might facilitate regulation of STICs and slow wave currents by NCX.

## Data Availability Statement

The datasets generated for this study are available on request to the corresponding author.

## Ethics Statement

The animal study was reviewed and approved by the Animal Use and Care Committee at the University of Nevada.

## Author Contributions

HZ planned, performed, and analyzed the electrophysiological experiments, collaborated on co-immunoprecipitation experiments, wrote the first draft of the manuscript, and edited the subsequent drafts. MZ performed and analyzed a portion of the electrophysiological experiments. BD and SB performed and analyzed Ca^2+^ imaging experiments. BP and YX planned and performed the co-IP and proximity ligation assays. KO’D generated clones and vectors for expression of ANO1 channels in HEK 293 cells. SK and KS conceived of the hypothesis, collaborated in planning experiments, analyzed the data, and wrote and edited the manuscript.

## Conflict of Interest

The authors declare that the research was conducted in the absence of any commercial or financial relationships that could be construed as a potential conflict of interest.

## Abbreviations

CaCC: Ca^2+^ activated Cl^–^ channels/conductance
ER: endoplasmic reticulum
FACS: fluorescence activated cell sorting
GI: gastrointestinal
ICC: Interstitial cells of Cajal
NCX: Na^+^/Ca^2+^ exchanger
NKCC1: Na-K-Cl cotransporter
PLA: proximity ligation assay
SMCs: smooth muscle cells
STD: spontaneous transient depolarization
STICs: spontaneous transient inward currents.
