# Corrigendum: Na^+^/Ca^2+^ Exchange and Pacemaker Activity of Interstitial Cells of Cajal

**DOI:** 10.3389/fphys.2020.00362

**Published:** 2020-04-15

**Authors:** Haifeng Zheng, Bernard T. Drumm, Mei Hong Zhu, Yeming Xie, Kate E. O'Driscoll, Salah A. Baker, Brian A. Perrino, Sang Don Koh, Kenton M. Sanders

**Affiliations:** Department of Physiology and Cell Biology, University of Nevada School of Medicine, Reno, NV, United States

**Keywords:** ANO1, Ca^2+^-activated Cl^−^ current, slow waves, smooth muscle, gastrointestinal motility

In the original article, we neglected to include the funding “National Institutes of Health, P20GM130459 to Haifeng Zheng.” The corrected Funding statement appears below.

The authors apologize for this error and state that this does not change the scientific conclusions of the article in any way. The original article has been updated.

